# Passive Radiative Cooling of Silicon Solar Modules with Photonic Silica Microcylinders

**DOI:** 10.1021/acsphotonics.2c01389

**Published:** 2022-11-08

**Authors:** Evelijn Akerboom, Tom Veeken, Christoph Hecker, Jorik van de Groep, Albert Polman

**Affiliations:** † Center for Nanophotonics, NWO-Institute AMOLF, Science Park 104, 1098 XGAmsterdam, The Netherlands; ‡ Department of Applied Earth Sciences, Faculty of Geo-Information Science and Earth Observation (ITC), University of Twente, Hengelosestraat 99, 7500 AAEnschede, The Netherlands; § Van der Waals-Zeeman Institute, Institute of Physics, University of Amsterdam, Science Park 904, 1098 XHAmsterdam, The Netherlands

**Keywords:** radiative cooling, photonics, Mie resonances, Kerker condition, antireflection coating, FTIR spectroscopy

## Abstract

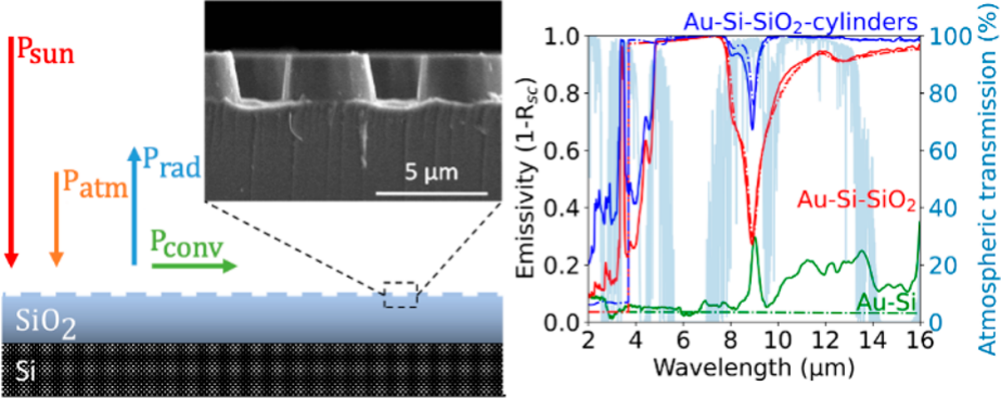

Passive radiative cooling is a method to dissipate excess heat from a material by the spontaneous emission of infrared thermal radiation. For a solar cell, the challenge is to enhance PRC while retaining transparency for sunlight above the bandgap. Here, we design a hexagonal array of cylinders etched into the top surface of silica solar module glass to enhance passive radiative cooling. Multipolar Mie-like resonances in the cylinders are shown to cause antireflection effects in the infrared, which results in enhanced infrared emissivity. Using Fourier transform infrared spectrometry we measure the hemispherical reflectance of the fabricated structures and find the emissivity of the silica cylinder array in good correspondence with the simulated results. The microcylinder array increases the average emissivity between λ = 7.5–16 μm from 84.3% to 97.7%, without reducing visible light transmission.

Over the past few decades, significant effort has been put into improving the power conversion efficiency of solar cells.^1^ The detailed balance limit^2^ calculates a fundamental efficiency limit of 29.7% for silicon-based solar cells,^3^ not far from the current record efficiency of 27.6%.^4^ Solar cell efficiency measurements are performed at standardized testing conditions, e.g., 1 sun illumination at an operating temperature of 25 °C. However, due to hot-carrier cooling and nonradiative recombination, a silicon solar cell typically reaches operating temperatures of 60 °C under direct sunlight, and even as high as 80 °C.^5^ Elevated operating temperatures reduce the power conversion efficiency and the operating lifetime of the cell. This efficiency reduction is mainly attributed to a decrease in the open-circuit voltage due to increased recombination rates.^6^ An average relative efficiency drop of −0.45% has been shown for every 1 °C temperature rise of mono- and poly crystalline silicon solar modules.^7^ A temperature increase from 25 to 60 °C amounts to a significant −15.75% efficiency drop. Even though the effects of elevated operating temperatures on the operating lifetime of a silicon solar module have not been isolated,^8^ it is expected to negatively impact all degradation modes.^9,10^ These adverse temperature effects emphasize the need for a method to cool Si solar modules. Here, we investigate the enhancement of passive radiative cooling (PRC) to decrease the operating temperature of a Si solar cell. The concept of PRC leverages the thermal emission of an object to dissipate heat to lower its temperature. According to thermodynamics, two objects with different temperatures will exchange heat via thermal radiation until an equilibrium temperature is reached. The Stefan–Boltzmann law states that the amount of heat emitted as thermal radiation scales with the temperature of an object as ∝*T*^4^, so effectively, heat is transferred from the warmer object to the colder object. Thus, to cool a hot object with thermal emission, a colder object is needed to function as the heat sink. Outer space is the perfect heat sink due to the temperature of about 3 K and the immense volume, which makes it a heat sink with practically infinite capacity. The concept of emitting thermal radiation into outer space is the core principle of PRC.

During the last two decades, interest in PRC has grown for several applications,^11^ from dew collection in remote and dry places^12^ to the cooling of buildings.^13^ In 2014, Raman et al. showed a 4.9 °C subambient daytime cooling using a thin-film multilayer to optimize the radiative properties of the structure.^14^ The multilayer was designed to enhance PRC while simultaneously excluding heating by reflecting incident solar radiation. However, the high reflectance of this geometry in the visible spectral range makes it particularly unsuitable for solar cell applications. A solar cell absorbs light with photon energy larger than the bandgap energy, including visible wavelengths.

Over the past years, several different materials and methods have been explored to cool solar cells with PRC: multilayers,^15,16^ 2D structures,^17−23^ a combination of a multilayer and 2D structures,^24,25^ or an effective medium approach.^26−28^Table 1 lists the calculated (italic) or fabricated (bold) material systems of a few notable works, and their reported temperature reduction: calculated values are in italic, measured values in bold font. To enable comparison of the achieved temperature reductions, it is important to specify the reference material system. This is particularly important since bare silicon exhibits practically no PRC, while a standard glass cover achieves significant PRC. Jaramillo-Fernandez et al. demonstrated the largest temperature reduction, both with respect to a bare silicon substrate and silicon covered with silica glass.^18^ They placed a self-assembled monolayer of silica spheres on top of the bare silicon and silicon-silica reference samples and measured an average temperature reduction of 14 and 9 degrees, respectively. Although the layer of spheres performs well, it may suffer from structural degradation in outdoor conditions. A second thing to be considered is the solar transmittance, since the silicon solar cell should be able to absorb visible light. Therefore, all discussed papers in Table 1 are using materials that are transparent in the visible wavelength range.

**Table 1:** Advances of Passive Radiative Cooling for Solar Cells^a^

first author (year)	passive radiative cooling structure	ref sample	temp reduction (K)
Zahir (2021)^16^	multilayer: *TiO_2_/BK7*	Si solar cell	*18.4*
		Si + SiO_2_	*5.4*
Perrakis (2021)^17^	2D structure: *SiO_2_ square micrograting with nanopillars on top*	Si	*5.8*
		Si + SiO_2_	*0.2*
Jaramillo-Fernandez (2019)^18^	2D structure: **self-assembly of SiO_2_ spheres**	Si	**14**
		Si + glass	**9**
Long (2019)^19^	2D structure: **SiO_2_ square lattice of microcylinders**	Si	*20*
		Si	**2**
Zhu (2015)^29^	2D structure: **lattice of air holes in SiO_2_**	Si	**13**
		Si + SiO_2_	**1**
Zhao (2018)^25^	multilayer + 2D structure: *TiO_2_/SiO_2_ with a lattice of air cylinders*	Si solar cell	*12*
		Si + SiO_2_	*8.3*
Chen (2021)^27^	effective medium: **SiO2 nanoparticles in polymer matrix**	Si solar cell	**5**
Akerboom (2022) [this work]	2D structure: **SiO_2_ microcylinder array**	Si	*21*
		Si + SiO_2_	*3*

a The calculated (italic) or fabricated (bold) PRC structure, the reference sample, and the calculated (italic) or measured (bold) temperature reduction are listed.

## Passive Radiative Cooling

**Figure 1:**
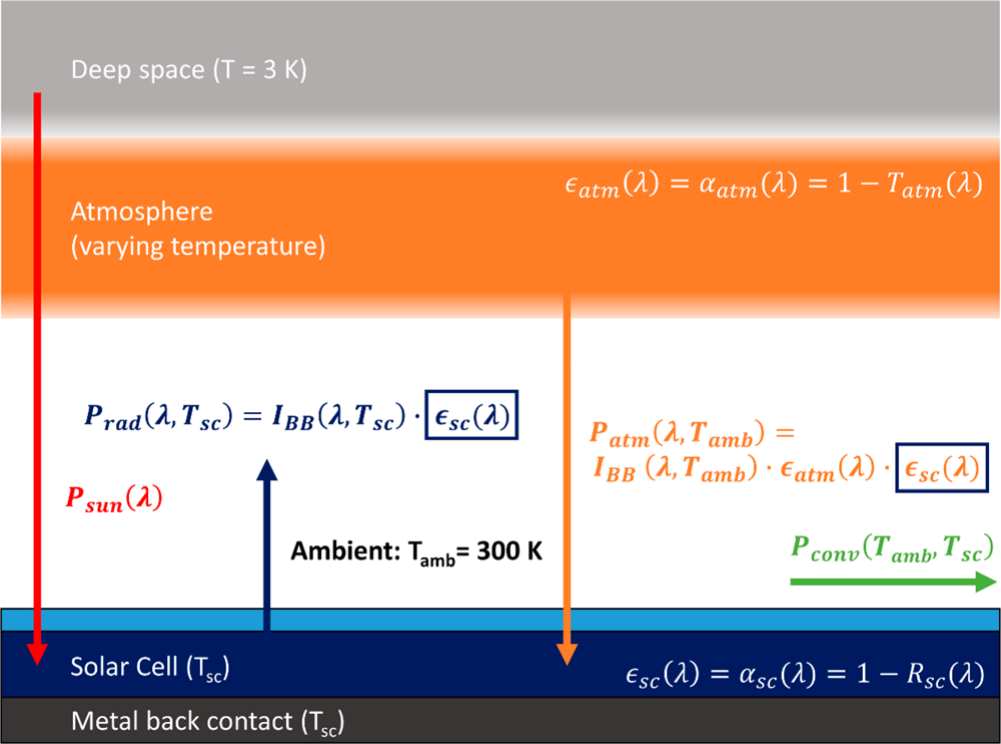
Schematic representation of the incoming and outgoing power flows that govern the equilibrium temperature of a solar cell (*T*_sc_). The solar cell reaches its equilibrium temperature when the power from the sun (*P*_sun_) and the thermal radiation from the atmosphere (*P*_atm_) are in balance with the thermal radiation emitted by the solar cell (*P*_rad_) and the power flow by convection and conduction (*P*_conv_).

The concept of passive radiative cooling (PRC) is based on the thermal balance of a solar cell. In Figure 1, a schematic representation of the energy balance of a solar cell is shown, indicating the four main power terms that determine the equilibrium temperature of the cell: the absorbed radiation coming from the sun (*P*_sun_), the absorbed thermal radiation from the atmosphere (*P*_atm_), the thermal radiation the solar cell is emitting (*P*_rad_), and the power gained by convection (*P*_conv_). Here we assume that heat conduction via connection to the rooftop is negligible. The total cooling power is given by the sum of the four main powers,
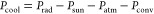
1

**Figure 2:**
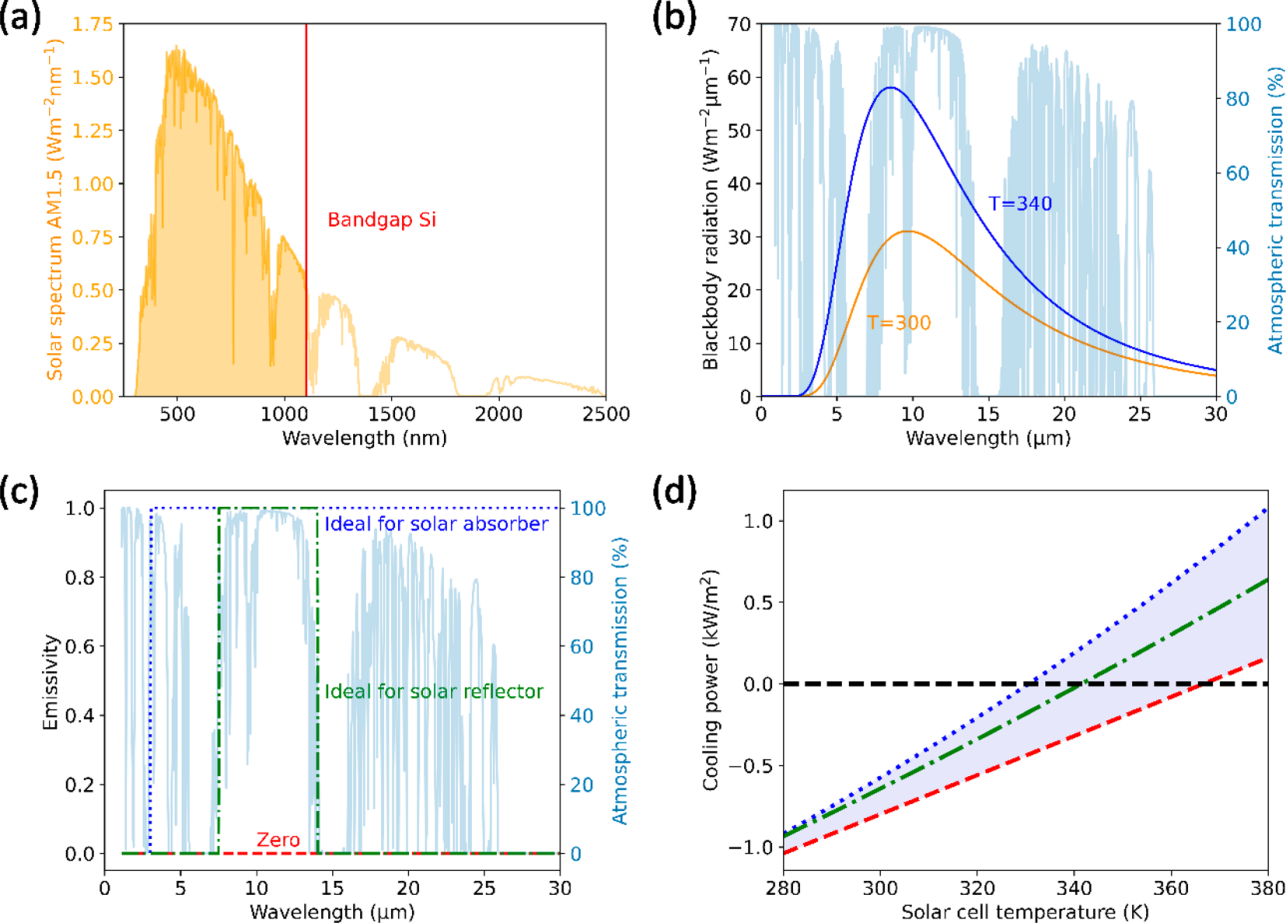
(a) AM1.5G solar irradiation spectrum (yellow line) as a function of wavelength, the bandgap of silicon (red line), and the part of the solar spectrum that can be absorbed by silicon (yellow surface). (b) Blackbody radiation spectra for an object at 300 K (orange) and 340 K (blue), and the atmospheric transmission (light blue). (c) Step function of three emissivity spectra: zero emissivity (red), a nonzero emissivity only in the main atmospheric transmission window (green), and the ideal emissivity for a body at 340 K (blue). (d) The calculated cooling power versus solar cell temperature (eq 1), corresponding to the emissivity spectra in (c). The equilibrium temperature is reached when the cooling power is zero.

When the total cooling power is zero, there is no net heat flux, and the solar cell has reached equilibrium temperature. A positive cooling power will effectively reduce the temperature, while a negative cooling power indicates the solar cell is heating up. Figure 1 shows a schematic representation of the power flows in eq 1; a detailed analysis of the interplay is provided in the Methods section. For a constant incident solar power *P*_sun_, integrated below silicon bandgap (Figure 2a), and *P*_conv_, we find that the cooling power is given by
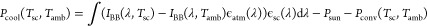
2Here, *T*_sc_ and *T*_amb_ are the temperatures of the solar cell and ambient, respectively, *I*_BB_ is the intensity of the blackbody spectrum, ε_atm_ is the emissivity of the atmosphere, and ε_sc_ the emissivity of the solar cell. To make sure that the first term in this equation is positive and contributes to cooling of the solar cell instead of heating, we can see that the emissivity of the solar cell (ε_sc_) must be zero when the incoming intensity from the atmosphere is larger than the intensity of the blackbody spectrum, and unity otherwise.

Figure 2b shows the blackbody spectra at ambient temperature (300 K) and a typical solar cell operating temperature (340 K). The spectrum of a nonideal blackbody is obtained by multiplying the ideal spectrum by the emissivity. By photonic engineering of the emissivity of the PRC layer, we can explore the effect of an emissivity spectrum on the resulting equilibrium temperature of the solar cell stack. In this comparison we assume that the PRC layer does not influence the absorbing properties of the solar cell in the visible wavelength range and take *P*_sun_ as a constant power corresponding to the integrated solar spectrum below silicon bandgap. First, we consider the upper temperature bound of zero emissivity for energies below the solar cell bandgap, plotted in Figure 2c in red. Calculating the cooling power with eq 1, we obtain the red curve in Figure 2d and an equilibrium temperature of 366.5 K. Second, we consider an emissivity window that is unity only in the main atmospheric transmission window between 8–14 μm, as shown in Figure 2c in green. This is the ideal emissivity spectrum for a solar reflector, enabling it to cool below ambient temperature by radiating through the atmosphere while keeping heat from the sun and atmosphere out. However, when used for a solar absorber material like a silicon solar cell, the resulting PRC is suboptimal with an equilibrium temperature of 341.5 K, as shown in Figure 2d in green. Third, we consider a solar absorber at a temperature higher than ambient. Due to this higher temperature, it emits more blackbody radiation than it receives from the atmosphere over the entire infrared wavelength range–see the intensity difference plotted in Figure 2b. Therefore, a solar absorber can achieve higher PRC by setting its emissivity to 1 throughout its entire blackbody radiation spectrum, from 3–30 μm, as plotted in blue in Figure 2c. Below 3 μm, the blackbody radiation at 340 K is negligible; thus, we set the emissivity to 0 between the silicon bandgap and 3 μm. This emissivity spectrum achieves the minimum equilibrium temperature at 330.5 K. We color the background of Figure 2d purple to indicate the attainable equilibrium temperatures to be used as a reference for the final results. In the next section, we look at the PRC of a silicon solar cell stack and improve the PRC with a photonic cylinder array.

## Photonic Design

In the previous section, we have derived a condition for the ideal emissivity of a solar cell: unity emissivity for wavelengths larger than 3 μm. The silicon solar cell itself has zero absorptivity (extinction coefficient) throughout the IR wavelength range (see Supporting Information, Figure S2), and thus zero emissivity (the reciprocity between absorptivity and emissivity according to Kirchhoff’s law is described in the Methods section). Therefore, we need to add a material to the solar cell to improve its PRC capacity, which is in thermal equilibrium with the solar cell. A typical solar module has a glass cover on top of the silicon, making it an obvious choice. Beyond 7.5 μm wavelength, the extinction coefficient off glass is nonzero, making them much more suitable materials for PRC than silicon (see Supporting Information, Figure S2 for the comparison of refractive index of abundant materials in PV).

**Figure 3:**
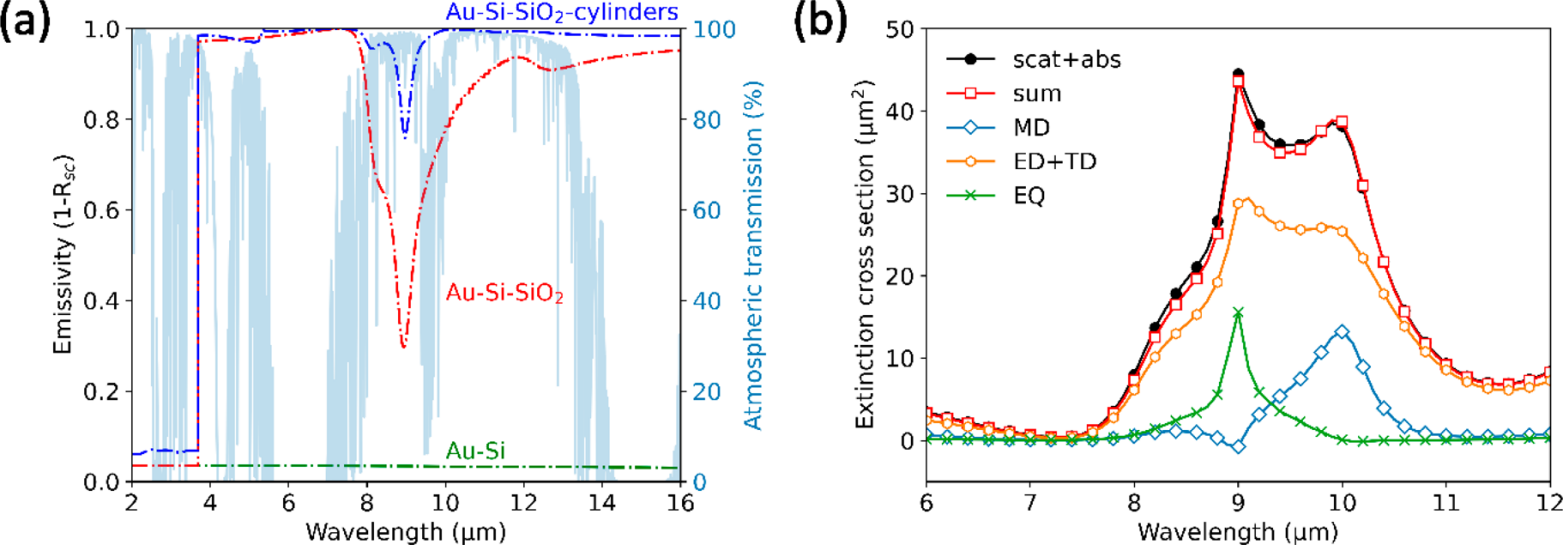
(a) Calculated emissivity of a stack of double-side polished silicon coated with gold (Au–Si), with a silica layer on top (red) and a silica layer (blue) with the optimized geometry of silica cylinders (radius of 1.75 μm, height of 2.25 μm and a pitch of 6.125 μm). Atmospheric transmittance is shown in light blue. The patterned module glass reduces and narrows the emissivity dip of glass at 9 μm, improving the radiative cooling of the stack. (b) Multipole decomposition for a silica cylinder (radius 1.75 μm, height 2.25 μm), where the contribution of several resonances to the total extinction cross-section is shown. The silica cylinder exhibits a broadband electric (ED) and toroidal dipole (TD), in combination with a narrower magnetic dipole (MD) and electric quadrupole (EQ).

We use a transfer matrix model (TMM, see Methods for details) to calculate the reflectivity of a solar module geometry consisting of a silicon substrate with a gold coating on the backside (solar cell back contact) and a quartz silica substrate on the top. The gold coating eliminates any transmission of light through this stack, and thus we can compute the emissivity as 1 – *R*_sc_, as shown in Figure 3a in red. The emissivity of the silica-on-silicon stack is much higher than the reference without silica, which is only slightly above zero due to parasitic absorption in the Au back coating (Figure 3a in green). The dip in the emissivity spectrum of the silica-on-silicon stack at 9 μm wavelength is caused by enhanced reflection at the air–silica interface. The enhanced reflection is a direct consequence of the strong fluctuation of the complex optical constants of silica in this spectral range, which is attributed to the asymmetric stretching vibration of Si–O–Si bridges.^30^ Even though we defined the ideal emissivity as unity until λ = 30 μm in the previous section, we carried out simulations until λ = 16 μm because this was the range of the experimental emissivity data.

In Table 1, we have already seen that significant calculated and measured temperature reductions have been achieved with 2D structures for enhanced PRC. However, only Zhu et al. made use of the solar cell module glass for their experimental PRC results, which would be ideal for practical reasons. They enhanced PRC by etching deep hollow cylinders, improving antireflection due to the gradual refractive index change.^29^ While this does enhance PRC, this approach does not include optimization for specific wavelengths. Therefore, we propose direct integration of a thinner 2D microstructure in the module glass, which we can optimize thoroughly. The microstructures should minimize the reflection between 7.5 and 16 μm wavelength (to minimize the dip in the red curve in Figure 3a) and simultaneously retain transparency for sunlight with photon energies above the silicon bandgap. To achieve antireflection, we design a hexagonal array of silica microcylinders on the silica substrate. These structures exhibit Mie-like resonances when their size is on the order of the wavelength.^31^ These types of resonant structures have received much attention in the field of nanophotonics.^32,33^ For photovoltaics, in particular, Mie-like resonant structures have been used to enhance light trapping,^34^ design solar cells with structural colors,^35^ and achieve antireflection for incident sunlight.^36,37^ Here, we design cylinders of several micrometers in size to achieve resonant antireflection for IR instead light. We use finite-difference time-domain (FDTD) simulations to optimize the dimensions of the hexagonal cylinder array (see Methods for technical details). We varied the cylinder diameter, height, and array pitch to minimize the reflection in the 4–16 μm wavelength range, finding the optimum for a diameter of 3.5 μm, a height of 2.25 μm, and a 6.125 μm pitch in a hexagonal array. The calculated emissivity is plotted in Figure 3a in blue, showing a significant increase compared to flat silica.

To gain an understanding of the resonant antireflection effect of the microcylinder array, we analyze the modal scattering contribution to the reflection spectrum. We attribute the antireflection effect to forward scattering of incident light by the multipolar Mie-like modes in the cylinders. According to the (generalized) Kerker condition, forward scattering is typically achieved by the interference of at least two different Mie-like modes.^38,39^Figure 3b shows the contributions of several Mie-like modes to the extinction cross-section of a single microcylinder, decomposed using the method outlined by Evlyukhin et al.^40^ The decomposition shows that the main broad contribution comes from the electric and toroidal dipoles (ED+TD). The magnetic dipole (MD) and electric quadrupole (EQ) contributions are slightly detuned from each other. When we consider the coherent excitation of these multipolar modes by a normal-incident plane wave, the modes oscillate in phase with each other. The symmetry of the modes leads to constructive interference between the ED and MD/EQ in the forward direction (transmission) and destructive interference in the backward direction (reflection). A “pure” Kerker condition of zero backscattering is achieved when the amplitude between the ED and MD/EQ are equal; this condition is typically only met for distinct wavelengths. Here, we achieve enhanced forward scattering by imperfect destructive interference of the ED and MD+EQ over a broad wavelength range. The comparison with Kerker-type inferences is shown in more detail in the Supporting Information. Figure S1 shows the polar scattering profile of an excited silica cylinder at 9 and 10 μm wavelength, and the polar emission profile of the interfering multipoles shown in Figure 3b. The resemblance of the profiles clearly indicates the Kerker-type forward scattering of our structure at these two wavelengths. By optimizing the shape of the resonator, it might be possible to further improve the destructive interference due to a better balance of the amplitudes of the modes. However, deviating from a radially symmetric shape also yields a polarization-dependent response. Moreover, destructive interference due to the interaction of multiple resonators or lattice modes is challenging with thermal sources,^41^ as we discuss next.

In general, interference between modes such as the Mie-like modes discussed above relies on a coherent phase-relation between them. However, the thermal emission that we consider as the source has only limited spatial coherence, typically on the order of λ/2.^42^ Between 8–16 μm wavelength, the coherence length would be 4–8 μm, which is larger than the size of the designed cylinder. That suggests that single-particle resonances such as the Mie-like modes, and their interference within the same particle, can be excited by thermal emission.

## Fabrication

**Figure 4:**
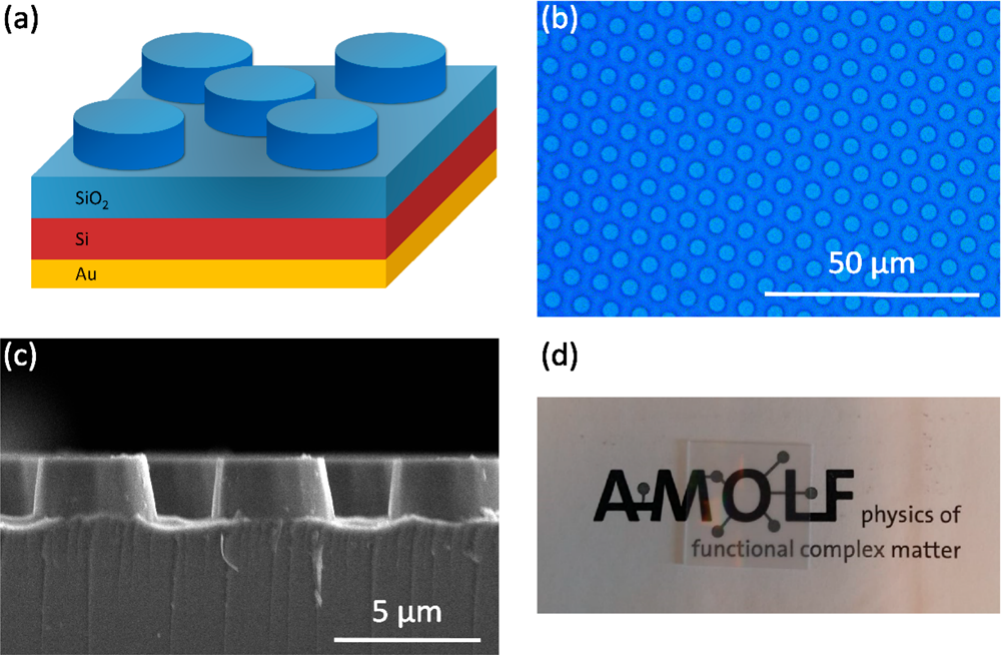
(a) Schematic representation of the hexagonal silica microcylinder array on top of a solar module stack. (b) Optical microscopy image of the array. (c) SEM image of a crosscut of the fabricated microcylinders, revealing straight, and slightly slanted sidewalls. (d) Photograph of the microcylinder array sample (24 × 24 mm^2^) on top of the AMOLF logo, showing high transparency in the visible spectral range.

Based on the theory and simulations of the previous section, we fabricate the hexagonal microcylinder array on top of a silica substrate. A double-side polished (DSP) silicon wafer with a gold coating on the back (see Methods) is placed under the silica to replicate a simple solar absorber (see Figure 4a). We used UV photolithography and subsequent reactive ion etching to realize the microcylinder array on top of a silica substrate (see Methods for details). An optical micrograph of the finalized array covering a 24 × 24 mm^2^ silica substrate is shown in Figure 4b, showing a uniform cylinder array and homogeneous color. Figure 4c shows a scanning electron microscope (SEM) image of a crosscut of the microcylinders, from which we determine a diameter of 3.65 μm and height of 2.20 μm, almost identical to the target dimensions of 3.50 (+4%) and 2.25 μm (−2.5%), respectively. The pitch is precisely 6.125 μm as designed. High transparency of the sample for visible light is visually demonstrated in Figure 4d, which shows a photograph of the sample on top of the AMOLF logo.

## Hemispherical Reflection Measurements

**Figure 5:**
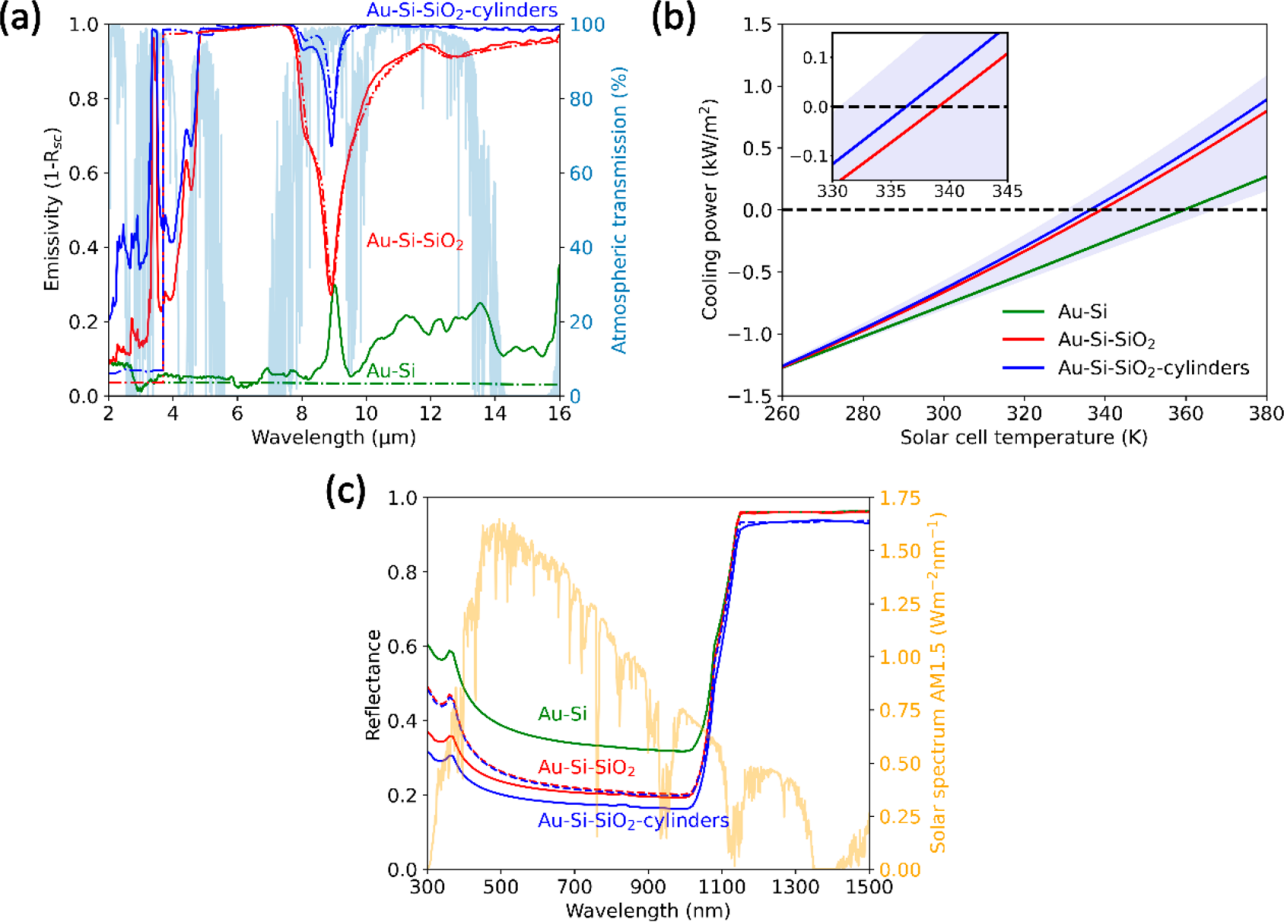
(a) Measured (solid) and calculated (dashed) IR emissivity of silicon without module glass (green), with flat silica module glass (red), and with microcylinder module glass (blue). The microcylinder array significantly increases the emissivity in the main atmospheric transmission window (light blue, 7.5–14 μm). (b) Calculated cooling power vs solar cell operating temperature corresponding to the measured emissivity in (a). The purple-colored background indicates the theoretical bounds, as calculated in Figure 3b. The inset shows a zoom-in of the calculated equilibrium temperatures (zero cooling power) for comparison with the theoretical minimum equilibrium temperature. (c) Measured visible to near-infrared hemispherical reflectance of bare silicon (green), covered with flat silica module glass (red) and covered with the microcylinder module glass (blue). The cylinder array lowers the reflectance for wavelengths below the silicon bandgap.

To characterize the IR emissivity of the fabricated microcylinder array sample, we measure the hemispherical reflectance in a Fourier-transform infrared (FTIR) spectrometer (see Methods). Figure 5a shows the calculated emissivity spectra (dashed, shown before in Figure 3a) and the experimentally obtained spectra (solid). The reference case of a bare double-side polished silicon substrate with a gold coating on the back is plotted in green. The calculated spectrum is almost zero (∼3.5%) because the extinction coefficient of Si is zero beyond λ = 1150 nm. However, the measured emissivity is significantly higher beyond λ = 9 μm, which we attribute to intraband transitions in the slight n-type doped silicon substrate.^43^ This small discrepancy does not influence our subsequent results because any IR light transmitted into the silica substrate is already absorbed before reaching the silicon substrate underneath. Thus, we can model the silicon to be nonabsorptive in the IR.

The measured emissivity of a flat silica substrate on top of a silicon substrate is shown in Figure 5a in red. An air gap between the silica and silicon substrates was avoided by adding a layer of immersion oil in between for the optical measurements. Beyond λ = 5 μm, the experimental emissivity is almost identical to the calculated spectrum. This result validates the experimental setup and the optical constants of silica that were used in the calculation. Moreover, this also validates that the thermal light source has the required coherence to excite the designed resonant modes: while we do not measure blackbody radiation, the excitation source is a thermal Globar source. The discrepancy between the calculated and experimental spectra in the range of 2–5 μm is attributed to a slight mismatch between the optical constants used for the calculation and the actual optical constants of the silica substrate. The absorption onset is quite abrupt for the literature values, just below 4 μm, while the actual values seem to gradually increase between 2–5 μm and exhibit more spectral features.

The measured emissivity of the silica substrate with the microcylinder array on top also resembles very well the calculated spectrum, as shown in Figure 5a in blue. Over a broad range from 7.5 to 16 μm, a significant increase in the emissivity was found, precisely as designed. The dip in the emissivity spectrum at 9 μm wavelength is reduced from 30% to 70%. Moreover, the average emissivity between 7.5 and 16 μm is increased from 84.3% to 97.7%.

Based on the experimental emissivity spectra, we now calculate the total cooling power as a function of operating temperature. Figure 5b shows three cooling power curves corresponding to the measurements in Figure 5a. The purple-colored background indicates the theoretical area between the upper and lower temperature bounds, as calculated in Figure 2d. At zero cooling power, we find the equilibrium temperature. As expected, we find that the silicon substrate with only the gold coating performs very poorly with an equilibrium temperature of 360 K, close to the maximum of 366.5 K. The addition of the flat silica substrate moves the curve closer to the minimum equilibrium temperature. The optimized silica microcylinder substrate pushes the equilibrium temperature even further toward the theoretical minimum. The flat silica stack and microcylinder silica stack reach equilibrium temperatures of 339 and 336 K, respectively, close to the minimum of 330.5 K. Interestingly, the flat silica substrate already achieves high PRC performance due to its near-ideal optical constants, realizing a cooling potential of 21 K compared to the bare silicon reference. The microcylinders decrease the equilibrium temperature by another 3 K. Assuming a 0.45% increase in efficiency per degree cooling, this temperature reduction would result in a relative efficiency increase of 1.35 and 10.8%, compared to the silicon solar module and the bare silicon reference, respectively. These values overestimate the equilibrium temperatures because they are based on experimental emissivity data up to λ = 16 μm (Figure 5a). In contrast, the theoretical maximum is based on unity emissivity until λ = 30 μm (Figure 2c).

The calculated equilibrium temperature corresponds well with the range of values listed in Table 1. An exact comparison of the reported temperature reductions is not possible because the calculation methods are not identical: the exact wavelength span and the optical constants vary. However, the results do show that choosing a proper module cover impacts the equilibrium temperature significantly. This is important for flat pane (silicon) solar cells, as discussed here, and concerns the development of flexible thin-film solar cells that use thin plastic module covers.

Finally, we perform hemispherical reflectance measurements in the visible to near-infrared (NIR) spectral range to verify that the fabricated microcylinder arrays do not adversely affect the transmission of sunlight into the solar cell for energies above the Si bandgap. Figure 5c shows the experimental reflectance for the two silicon-silica stacks. The reflectance is around 20% up to the silicon bandgap, mostly due to reflection at the silica-silicon interface. Beyond 1100 nm, the reflectance is higher because the gold coating at the back reflects most NIR light. This is significantly higher than the reflection of commercial solar modules, which achieve only a few percent reflection due to antireflection coatings and/or textures. The reflection could be decreased for all three cases by adding an AR coating on top of the silicon. The comparison in Figure 5c shows that the microcylinders slightly decrease the reflection of light for wavelengths smaller than the silicon bandgap. This difference is explained by light trapping: light is scattered by the microcylinders and trapped in the silica substrate by total internal reflection.

## Conclusions

This work shows that the passive radiative cooling (PRC) power of a silicon solar module can be enhanced by placing an array of microcylinders on top of the module glass. Photonic Mie-like resonances in the silica cylinders reduce infrared (IR) light reflection at the silica-air interface through engineered destructive interference of the resonant multipolar modes. By reciprocity, this improved antireflection effect increases the IR emissivity of the silica module glass. First, we studied the optimal emissivity profile for a typical silicon solar module that operates at elevated temperatures. By examining the thermal balance of a solar cell at *T* = 340 K, we found quartz silica to be the ideal module glass material due to its broad extinction coefficient in the λ = 3–30 μm spectral range. Subsequently, we designed a microcylinder array etched into the silica and optimized the dimensions for enhanced emissivity with FDTD simulations. The cylinder array is optimized to reduce the emissivity dip around λ = 9 μm that is caused by strong reflection at the silica-air interface.

Next, the microstructures were fabricated by UV photolithography and reactive ion etching into a silica substrate. To mimic a silicon solar module, we placed the microstructured silica substrate on a silicon substrate with a gold coating on the back. This stack has zero transmittance throughout the visible and IR wavelength ranges. The measured hemispherical IR reflectance compares very well to the simulated results, demonstrating the designed PRC enhancement. The fabricated microstructure increased the average emissivity between λ = 7.5–16 μm from 84.3% to 97.7%. Moreover, the microstructured silica substrate shows a slight decrease in reflectance in the spectral range where the Si solar cell absorbs. In conclusion, the flat silica coating already achieves high PRC due to its near-ideal optical constants, realizing a cooling potential of 21 K compared to the bare silicon reference. The microcylinders decrease the equilibrium temperature by another 3 K. Assuming a 0.45% increase in efficiency per degree cooling, this would result in a relative efficiency increase of 1.35–10.8%, compared to the silicon solar module and the bare silicon reference, respectively. This insight is also relevant for the development of lightweight photovoltaics that do not use a glass cover.

These results highlight the opportunities of thermal management for photovoltaic applications by considering the module glass as an integral part of the photonic design. Our design concepts are general and applicable to all solar cell designs as well as module glass materials. The recent advancement of optimization algorithms and complex shape fabrication by 3D printing and conformal imprinting could improve the cooling potential of the design even further.

## Methods

### Passive Radiative Cooling Calculation

In Figure 1, a schematic representation of the energy balance of a solar cell is shown, indicating the four main power terms that determine the cooling power of the cell:
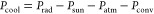
M1Here, *P*_sun_ is the absorbed radiation coming from the sun, *P*_atm_ the absorbed thermal radiation from the atmosphere, *P*_rad_ the thermal radiation the solar cell is emitting, and *P*_conv_ the power lost or gained by convection. We assume that heat conduction via connection to the rooftop is negligible.

The primary energy input is the irradiation from the sun, which is for normal incidence given by
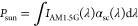
M2with *I*_AM1.5G_ the solar irradiation within AM1.5G^44^ and α(λ) the absorptivity of the solar cell. In Figure 2a, the AM1.5G solar spectrum is shown, and the silicon bandgap energy is indicated. The AM1.5G solar spectrum has an integrated power of 1000 Wm^–2^, but silicon does not absorb light with energies below its bandgap. Therefore, for further calculations, *P*_sun_ is set to 808 Wm^–2^, which is the integrated power in the AM1.5G solar spectrum for energies above the silicon bandgap (the yellow surface in Figure 2a). Effectively, the emissivity of the stack is unity for wavelengths below 1.1 μm (this is not shown in Figure 2c). The power input from the sun is completely independent of the infrared emissivity that is tuned to improve PRC. Therefore, this factor influences the absolute equilibrium temperatures, but not the slope of the temperature curves in Figures 2d and 5b, nor the distance between different curves (see Supporting Information, Figure S3). For this comparative study, the precise value of *P*_sun_ is thus unimportant. This also allows us to neglect the fact that a solar cell converts about 20% of the incoming solar power into electricity rather than heat.

Second, heat can be exchanged between the solar cell and its environment through convection, given by the product of the nonradiative heat transfer coefficient (*h*_c_) and the temperature difference between the solar cell and the ambient environment:
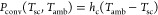
M3

We set the nonradiative heat transfer coefficient to 6 Wm^2–^ K^–1^, corresponding to a wind speed of 1 m/s.^21^

The solar cell radiates as a nonideal blackbody, so its emitted radiative power is given by
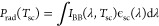
M4which is the product of the emissivity of the solar cell (ε_sc_(λ)), a number between zero and one that determines the quality of the solar cell as a blackbody, and the blackbody radiation according to Planck’s law:^45^
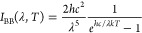
M5

The atmosphere also radiates as a nonideal blackbody. The radiative power from the atmosphere that is absorbed by the solar cell is given by
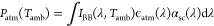
M6which is the product of the blackbody radiation from the atmosphere, the emissivity of the atmosphere (ε_atm_(λ)), and the solar cell absorptivity (α_sc_(λ)). The blackbody spectrum of the atmosphere depends on the ambient temperature, *T*_amb_, just as the blackbody spectrum of the sun depends on the temperature of its surface.

Then we use Kirchhoff’s law for a body in thermal equilibrium, which states that the emissivity equals the absorptivity at every wavelength:
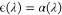
M7

Substituting the absorptivity for the emissivity in eq M6, we find that the cooling power depends on the balance between incoming radiation from the atmosphere and the outgoing blackbody radiation from the cell:
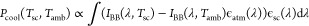
M8

Here, the emissivity of the atmosphere at normal incidence^21^ is given by
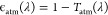
M9where *T*_atm_(λ) is the atmospheric transmittance.^46,47^

Both terms in eq M8 depend on the emissivity spectrum of the solar cell, ε_sc_(λ), which is the most important parameter to tune to achieve PRC. The integrand in eq M8 scales linearly with ε_sc_(λ), so the radiative cooling power can readily be optimized by calculating the integrand for each wavelength λ_*i*_, at a given solar cell and ambient temperature. If the integrand is positive, that is, at λ_*i*_ the solar cell emits more radiation than it receives from the atmosphere, the ideal emissivity ε_sc_(λ_*i*_) = 1:
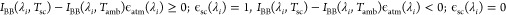
M10

In the case of a solar reflector, a body with a temperature equal to the ambient temperature (*T*_sc_ = *T*_amb_), this criterion sets ε_sc_(λ_*i*_) = 1 only for low atmospheric emissivity. This ideal curve is plotted in Figure 2c in green, where the emissivity is 1 only in the main atmospheric transmission window between 8–14 μm.

In the case of a solar absorber like a silicon solar cell, the operating temperature is higher than the ambient temperature (*T*_sc_ > *T*_amb_). Figure 2b shows the atmospheric transmission with the blackbody spectra at temperatures 300 and 340 K, corresponding to ambient temperature and the approximated temperature of an operating solar cell, respectively. Thus, the solar absorber emits more blackbody radiation than it receives from the atmosphere, independent of the atmospheric emissivity. Therefore, the ideal emissivity of a solar absorber is unity between λ = 3–30 μm, as plotted in blue in Figure 2c. Below 3 μm, there is negligible blackbody radiation at 340 K, so we set the ideal solar absorber emissivity to 0 between λ = 3 μm and the solar cell bandgap.

The emissivity of the solar cell also equals its absorptivity (Kirchhoff’s law, eq M7), which can be determined by
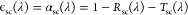
M11with *R*_sc_(λ) as the reflection and *T*_sc_(λ) as the transmission of the solar cell. Maximizing the IR emissivity of the solar cell thus equals minimizing the reflection and transmission.

### Transfer Matrix Model

We used a transfer matrix model based on the Fresnel equations to calculate the reflection and transmission of planar multilayer stacks. In particular, we used the Python implementation written by Steven J. Byrnes.^48^ Literature values for the optical constants of Si and SiO_2_ (Supporting Information, Figure S2) were used from ref (49) and for Au from ref (50).

### FDTD Simulations

The optimization of the microcylinder array was performed by finite-difference time-domain (FDTD) calculations using Lumerical FDTD solutions.^51^ The single-pass IR reflection was minimized for a hexagonal array of silica cylinders at the interface of a semi-infinite silica substrate and air superstrate. Minimizing the reflection led to maximizing the cooling power (see the calculation above), assuming that all IR light transmitted into the silica substrate is absorbed. The cylinder array was simulated in periodic boundary conditions. Convergence was found for a uniform 25 nm mesh size, conformal mesh refinement, and 10^–7^ auto shutoff value. With a brute-force optimization procedure the optimized design was found to be a hexagonal array of silica cylinders, height 2.25 μm and radius 1.75 μm, with a constant pitch at 3.5 times the radius. The results of the optimization procedure are shown in Supporting Information, Figure S4. The figure of merit is the radiative part of *P*_cool_, according to eq M8, integrated between λ = 2–16 μm.

The multipole decomposition in Figure 3b was performed by calculating the electric field inside the microcylinder according to the method outlined by Evlyukhin et al.^52^

### Electron-Beam Physical Vapor Deposition

A double-side polished (DSP) silicon wafer (WRS Materials, lightly phosphorus n-type doped, resistivity 1–20 Ωm) with a thickness of 500 μm was used as the absorber substrate. Electron-beam physical vapor deposition was used to deposit an 80 nm gold layer on one side of 24 × 24 mm^2^ DSP Si substrates at a deposition rate of 0.5 Å/s.

### UV Photolithography and Reactive Ion Etching

As a photolithography mask, a negative photoresist, ma-N 1420, was spin coated onto a 4 in. silica wafer of 500 μm thickness. A hexamethyldisilazane (HMDS) resist adhesion promotor was spin coated on the wafer at 4000 rpm with 1000 rpm/s for 35 s, followed by a curing step on a 150 °C hot plate for 1 min. A 2 μm thick layer of ma-N 1420 was spin coated at 2000 rpm with 500 rpm/s for 30 s and cured at 100 °C for 2 min.

The photoresist was illuminated (λ = 365 nm) in a UV mask aligner (Süss MicroTec MABA6) through a quartz substrate with a chrome mask (commercial, Delta Mask BV). Unwanted interference due to reflection from the bottom silica-air interface is decreased by placing an absorptive tape on the back. The resist was developed by immersing the wafer into ma-D 533/S photoresist developer for 75 s; the wafer was then rinsed in H_2_O for 30 s (twice) and blow-dried using a nitrogen gun.

The microstructured photoresist was used as a reactive ion etching (RIE) mask in an Oxford PlasmaPro Cobra RIE. A plasma of 50 sccm C_4_F_8_ and Ar gases was used to etch 2.20 μm deep in 16:30 min. The remaining photoresist was removed by immersion in base piranha, rinsed in H_2_O, and dried under a nitrogen gun. Finally, the wafer is cut into 24 × 24 mm^2^ substrates.

### Infrared Hemispherical Reflectance

The infrared hemispherical reflectance measurements were conducted in a modified Bruker Vertex70 research-grade laboratory Fourier-transform infrared (FTIR) spectrometer at the University of Twente, as described in work by Hecker et al.^53^ The Vertex70 is modified with an external, custom-made integrating sphere with a diffuse gold coating, with a Mercury Cadmium Telluride (MCT) infrared detector on top (λ = 1–16.7 μm). The sample is positioned at the south pole of the sphere, with a variable aperture set to 20 mm. An external, high-power Globar source (λ = 1–16.7 μm) was used for high signal-to-noise measurements.

Measurements are taken at a wavenumber resolution of 8 cm^–1^ and repeated 8 times to average the noise. All measurements are normalized to the reflection of a diffuse or flat gold substrate, and the signal of an open sample port measurement is subtracted as background. The silica substrates were attached to the silicon substrate with immersion oil (Honeywell 10976) to avoid an air gap in between the substrates, which would cause unwanted Fabry–Perot resonances. The immersion oil was checked to be transparent throughout the visible-NIR wavelength range until the silicon bandgap. Moreover, the transmittance of the silica substrate is 0 throughout the IR, so the immersion oil will not influence the FTIR measurements.

### Visible to near-Infrared Hemispherical Reflectance

Hemispherical reflectance measurements were conducted in the visible to near-infrared (NIR) wavelength ranges with a PerkinElmer LAMBDA 750 UV/vis/NIR. A deuterium and a tungsten lamp were used, in combination with a double holographic grating monochromator to illuminate the sample with monochromatic light. The sample was placed at the back of a 150 mm integrating sphere at an angle of 8°, and the hemispherical reflectance was detected using a PMT and an InGaAs detector for the wavelength ranges 300–860 nm and 860–2500 nm, respectively. The integration time was 0.2 s, and the signal was averaged over three times to increase the signal-to-noise ratio.

## Supporting Information Available

Figures of the simulated far-field radiation of the designed structures which show the forward scattering, a comparison between the refractive index in the IR for several abundant materials in PV, a calculation of the impact of solar power and ambient temperature on the equilibrium temperature of the solar cell, and the brute force optimization showing the robustness of the photonic design (PDF)
