# Supplementary material for: Passive Radiative Cooling of Silicon Solar Modules with Photonic Silica Microcylinders
Source: ACS Photonics. 2022 Nov 8;9(12):3831–40. doi: 10.1021/acsphotonics.2c01389 (PMC9782778; doi:10.1021/acsphotonics.2c01389)
Supplement: Supplementary file 1 — ph2c01389_si_001.pdf [file ph2c01389_si_001.pdf]

Passive radiative cooling of silicon solar modules with photonic silica microcylinders

Evelijn Akerboom,[*,#,a] Tom Veeken,[#,a] Christoph Hecker,[b] Jorik van de Groep,[c] and Albert Polman[*,a]

[#] Evelijn Akerboom and Tom Veeken contributed equally to this work.

* Corresponding authors: e.akerboom@amolf.nl, a.polman@amolf.nl

[a] Center for Nanophotonics, NWO-Institute AMOLF, Science Park 104, 1098 XG Amsterdam, the Netherlands

[b] Department of Applied Earth Sciences, Faculty of Geo-Information Science and Earth Observation (ITC), University of Twente, Hengelosestraat 99, 7500 AA Enschede, The Netherlands

[c] Van der Waals-Zeeman Institute, Institute of Physics, University of Amsterdam, Science Park 904, 1098 XH Amsterdam, The Netherlands

---

Content

S1: Simulation of polar emission profiles of the structures

S2: Comparison of refractive indices for prevalent materials in PV

S3: Calculation of the equilibrium temperature of the solar cell for relevant solar power and ambient temperature conditions

S4: Brute force optimization of our designed structures

Supporting Information

Kerker conditions photonic design

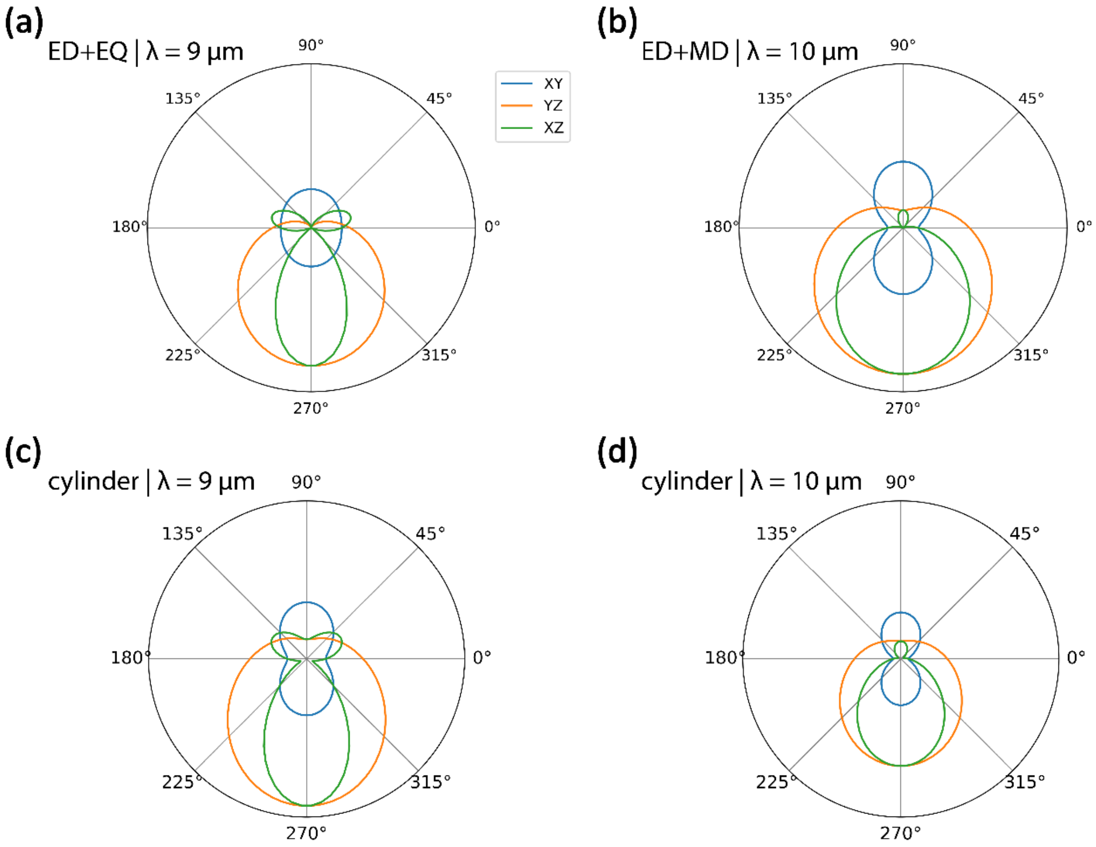

Figure S1: ***(a)*** *Polar emission profile of the interference between an electric dipole (ED) and an electric quadrupole (EQ) and* ***(b)*** *polar emission profile of the interference between the electric dipole (ED) and the magnetic dipole (MD) (dipole amplitudes are weighted according to the multipole decomposition in Figure 3b). These forward emission profiles are close to the ideal generalized Kerker profiles.* ***(c)*** *and* ***(d)*** *the polar scattering profile from a single glass cylinder excited at 9 µm and 10 µm wavelength, respectively. The scattering profiles resemble the Kerker-like emission profiles in (a) and (b), indicating that the interference of Mie-like modes causes the designed anti-reflection. [The results were computed using FDTD calculations: in (a) and (b), electric and magnetic dipole sources were used, and in (c) and (d), a TFSF plane-wave source was used. In both cases, the emitted/scattered radiation was computed in a power monitor box. Lumerical's built-in near-to-farfield transform was used to obtain the far-field profiles* [1].

Refractive index dielectric materials

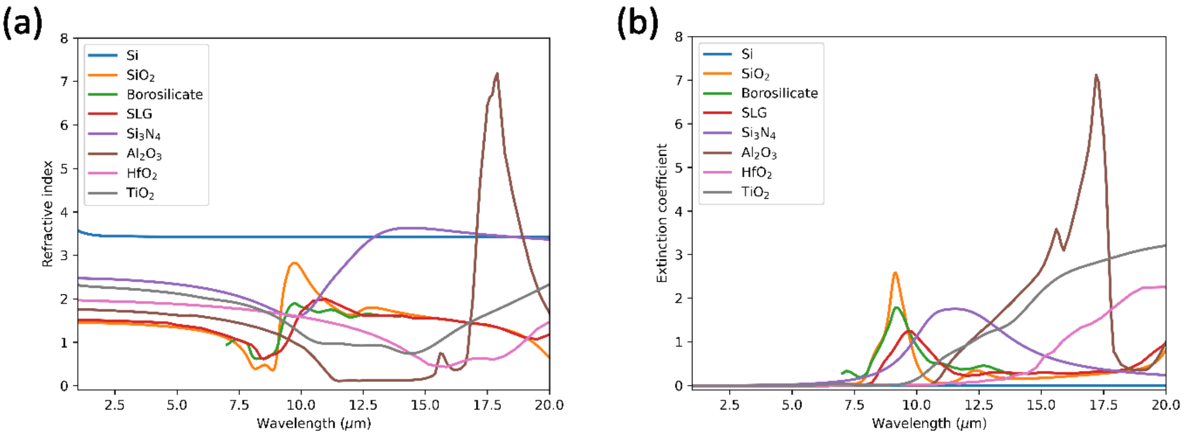

Figure S2: ***(a)*** *The refractive index and* ***(b)*** *the extinction coefficient of the most prevalent materials in PV: Si* [2], SiO_2 [2], *Borosilicate* [3], *Soda lime glass (SLG)* [4], *Si_3N_4* [5], *Al_2O_3* [2], *HfO_2* [6] *and TiO_2* [7].

Equilibrium temperature of the solar cell

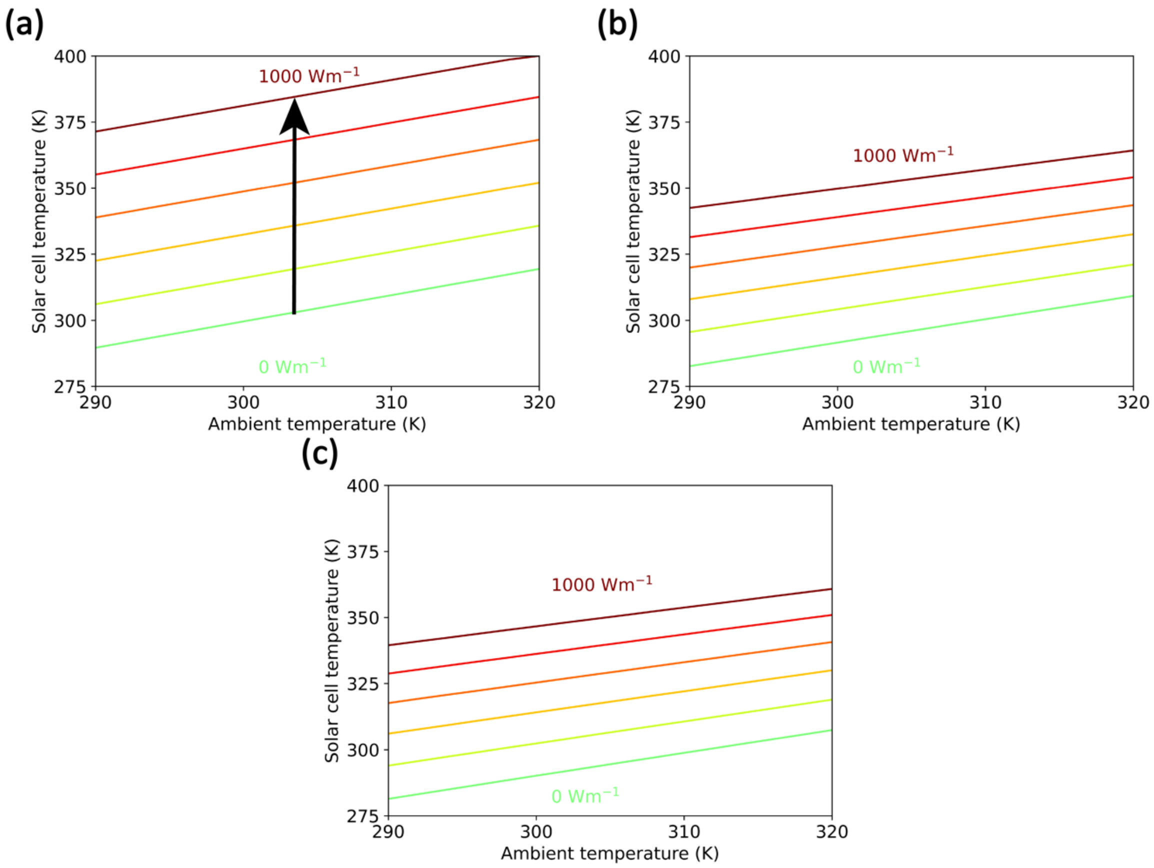

Figure S3: *The equilibrium temperature of the solar cell for different conditions of ambient temperature and incoming solar power for* ***(a)*** *the bare silicon,* ***(b)*** *the silicon with a flat silica module glass, and* ***(c)*** *the microcylinder module glass. The result shows that the equilibrium temperature scales linearly with both the ambient temperature and the solar power.*

Design optimization

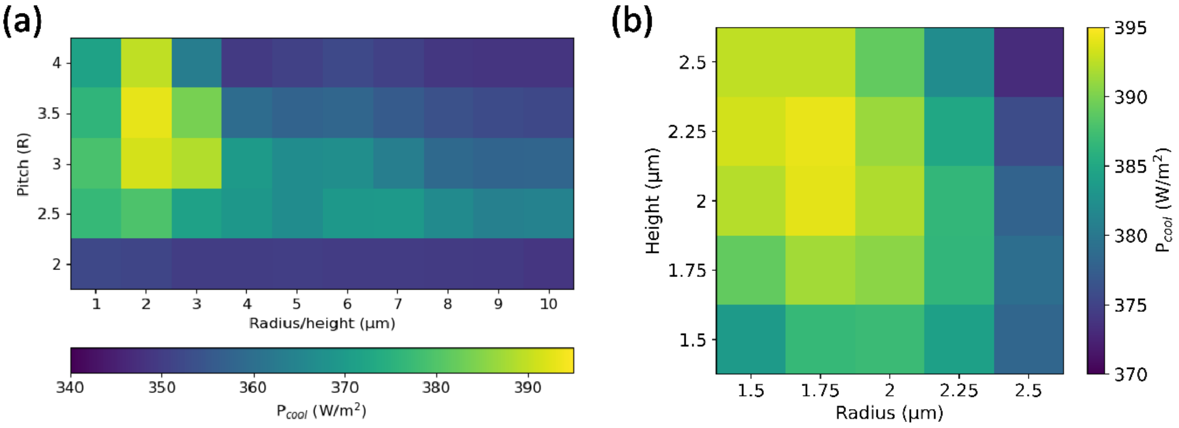

Figure S4: *Optimization of the hexagonal microcylinder array.* ***(a)*** *Pitch optimization as a function of radius = height; maximum at pitch = 3.5 × radius.* ***(b)*** *Radius and height optimization, using optimal pitch found in (a). Optimal geometry was found at radius = 1.75 µm, height = 2.25 µm, and pitch = 3.5 × 1.75 = 6.125 µm. Color scaling is calculated according to Eq. (1), with 808* Wm^{-2} *solar power at 300 K ambient temperature.*

References

(1) ANSYS Lumerical. FDTD Solutions. 2022.

(2) Palik, E. D. *Handbook of Optical Constants of Solids, Author and Subject Indices for Volumes I, II, and III*, first.; Academic Press, 1997; Vol. 1.

(3) Luo, J.; Smith, N. J.; Pantano, C. G.; Kim, S. H. Complex Refractive Index of Silica, Silicate, Borosilicate, and Boroaluminosilicate Glasses – Analysis of Glass Network Vibration Modes with Specular-Reflection IR Spectroscopy. *J Non Cryst Solids* **2018**, *494* (April), 94–103. https://doi.org/10.1016/j.jnoncrysol.2018.04.050.

(4) Rubin, M. Optical Properties of Soda Lime Silica Glasses. *Solar Energy Materials* **1985**, *12* (4), 275–288. https://doi.org/10.1016/0165-1633(85)90052-8.

(5) Kischkat, J.; Peters, S.; Gruska, B.; Semtsiv, M.; Chashnikova, M.; Klinkmüller, M.; Fedosenko, O.; Machulik, S.; Aleksandrova, A.; Monastyrskyi, G.; Flores, Y.; Masselink, W. T. Mid-Infrared Optical Properties of Thin Films of Aluminum Oxide, Titanium Dioxide, Silicon Dioxide, Aluminum Nitride, and Silicon Nitride. *Appl. Opt.* **2012**, *51* (28), 6789–6798. https://doi.org/10.1364/AO.51.006789.

(6) Bright, T. J.; Watjen, J. I.; Zhang, Z. M.; Muratore, C.; Voevodin, A. A. Optical Properties of HfO 2 Thin Films Deposited by Magnetron Sputtering: From the Visible to the Far-Infrared. *Thin Solid Films* **2012**, *520* (22), 6793–6802. https://doi.org/10.1016/j.tsf.2012.07.037.

(7) Siefke, T.; Kroker, S.; Pfeiffer, K.; Puffky, O.; Dietrich, K.; Franta, D.; Ohlídal, I.; Szeghalmi, A.; Kley, E. B.; Tünnermann, A. Materials Pushing the Application Limits of Wire Grid Polarizers Further into the Deep Ultraviolet Spectral Range. *Adv Opt Mater* **2016**, *4* (11), 1780–1786. https://doi.org/10.1002/adom.201600250.
